# The Use of an Artificial Intelligence-Driven Novel Tool for the Evaluation of Dental Implants Primary Stability and Immediate Loading Feasibility: A Multicenter Retrospective Study

**DOI:** 10.3390/jcm14062011

**Published:** 2025-03-16

**Authors:** Marco Degidi, Giuseppe Daprile, Filippo Battelli, Ernesto Caselli, Luca Cisternino, Alessandro Greco, Daniele Palumbo, Federico Quasso, Fabio Rossi, Corrado Tavelli, Zoran Zaccheroni

**Affiliations:** 1 Private Practice, 40100 Bologna, Italy; gdaprile@libero.it (G.D.);; 2 Private Practice, 47921 Rimini, Italy; 3 Private Practice, 60121 Ancona, Italy; 4 Private Practice, 25121 Brescia, Italy; 5 Private Practice, 22100 Como, Italy; 6 Private Practice, 20831 Seregno, Italy; 7 Private Practice, 23100 Sondrio, Italy; 8 Private Practice, 40026 Imola, Italy

**Keywords:** primary stability, artificial intelligence, dental implants, immediate loading, insertion torque

## Abstract

**Background/Objectives:** A novel tool based on artificial intelligence (AIT) to evaluate immediate loading feasibility was recently introduced. The aim of this study is to evaluate the correspondence between the AIT prediction and the operator’s evaluation in a large sample of implants. **Methods:** 11 operators were asked to classify the recorded insertion curves of all the implants placed during the period between September 2022 and August 2023 as suitable or non-suitable for immediate loading. Next, the same curves were analyzed by the AIT, which classified them as belonging to YES (suitable for immediate loading) or NO (non-suitable for immediate loading) class. **Results:** 1320 dental implants were placed and a total of 21 different implant systems were used. According to the surgeons’ evaluation, 999 curves were suitable and 321 were non-suitable for immediate loading; when evaluated by the AIT, 916 curves belonged to class YES, while 404 belonged to class NO. The resulted sensitivity was 90.49% (95% CI = 88.5% to 92.2), specificity was 96.26% (95% CI = 93.6% to 98.1%), PPV was 98.7% (95% CI = 97.7% to 99.3%), and NPV was 76.5% (95% CI = 72% to 80.5%). **Conclusions:** The AIT tested in the present clinical multicenter study demonstrated a high level of accuracy in the prediction of immediate loading feasibility.

## 1. Introduction

In recent decades, the use of immediate loading techniques has rapidly spread, it is now a routine procedure. Especially with these techniques, implant primary stability is a fundamental prerequisite [1] as well as a proven system for measurement.

Primary stability can be defined as the biometric stability of an implant directly after its insertion and is mainly obtained by the contact between the bone and the fixture [2]. Primary stability is essential for the deposition and mineralization of bone on the implant surface [3] and is influenced by several variables: length, diameter, geometry, surface of the implant, bone density, and site preparation technique [3,4].

In the past, implant primary stability was evaluated exclusively by means of surgeons’ perception, but the method was affected by an important personal experience bias and presented a high error rate [5]. By consequence, a few systems were introduced to overcome the problem and to acquire an unbiased evaluation; in particular, peak insertion torque (pIT) and Resonance Frequency Analysis (RFA) were largely used worldwide [6]. Peak insertion torque is recorded by means of a torque gauge housed in the surgical motor, or by using manual wrench rachets, which are susceptible to wear and, consequently, can become imprecise over time [7]. RFA is measured only by a specific electronic device and a transducer secured to the implant by a screw; nevertheless, its sensitivity in primary stability evaluation is questionable [1].

These two methods are also utilized in combination to obtain a more precise indication; nevertheless, it was demonstrated in [8] that RFA and pIT measure two different qualities of primary stability. Thus, the two measurements can show opposing results and can cause clinician misinterpretation, which can affect the immediate loading feasibility decision.

In 2013, Variable Torque Work (VTW) was presented [9]; this new method involved the calculation of the area underlying the insertion torque curve recorded during implant insertion. It displays the insertion torque produced during the insertion with more accuracy and it does not show only the maximum or the ending values.

As an evolution of the concept, a novel tool based on artificial intelligence (AIT) was introduced and described in detail in a recently published paper [10]. AIT can classify insertion torque curves with different trends and lengths into two categories, defined as “YES” and “NO.” The “YES” category corresponds to curves suitable for immediate loading, and the “NO” class to a non-suitable curve. The categorization is performed by a supervised algorithm that relies on samples of already read insertion torque curves classified as S (suitable) or NS (non-suitable) for immediate loading. The AI model chosen was a convolutional neural network (CNN). In comparison to other artificial neural network models, this specific one is very fast in training and prediction, is not influenced by stability problems during the training phase, and has a high prediction power.

In the publication, an in vitro pilot study to evaluate the level of accuracy in the prediction of immediate loading suitability of the method was also presented: five different implant geometries and a single operator were involved. The authors showed that the correspondence between surgeons and the AIT evaluation was 99.3%, with only one false negative reported by the algorithm analysis. The sensitivity and the specificity were, respectively, 98.95% and 100%. Nevertheless, the study was conducted by a single testing clinician who was involved in the training phase of the algorithm; furthermore, the sample was limited and the implants were placed in polyurethane blocks. For all these reasons, these very high levels of accuracy should be interpreted with caution.

The aim of this clinical study is to evaluate the correspondence between AIT prediction and operators’ evaluation in a larger sample with a greater number of implant geometries and surgeons involved.

## 2. Materials and Methods

For this study, 11 operators with at least 10 years of expertise in immediate loading techniques were recruited. Each surgeon was asked to analyze the insertion torque curves, anonymously recorded by means of a specific surgical motor (iChiropro; Bien-Air Dental SA, Bienne, Switzerland), of all implants inserted during the period between September 2022 and August 2023, with no limitations in terms of implant geometry and dimensions, surgical technique, or bone density. The operators were asked to classify the insertion curves as suitable or non-suitable for immediate loading, considering every implant as presumed to be immediately loaded by a single non-splinted crown. Next, the same curves were analyzed by the new AIT, which classified them as belonging to YES (suitable for immediate loading) or NO (non-suitable for immediate loading) classes [10]. In order to limit excessive differences, only the first 500 implants placed by a single operator were taken into consideration. The study was conducted in accordance with Helsinki declaration of 1975 as revised in 2008 and insertion torque curves were recorded with the patients’ informed consent.

### Statistical Analysis

For the whole sample, sensitivity, specificity, positive predictive value (PPV), negative predictive value (NPV), receiver operating characteristic curve (ROC), and area under the ROC curve (AUC) were assessed. A *p*-value <0.05 was considered significant and 95% confidence intervals (95%CI) were calculated.

Due to the large variability in implant geometries and clinical situations included in the final sample, it was not possible to conduct an inter-rater reliability test. Nevertheless, in order to evaluate possible differences between operators, sensitivity, specificity, positive predictive value (PPV), and negative predictive value (NPV) tests were performed for the surgeons that placed at least 50 implants during the study period.

Statistical analysis was performed by means of MedCalc for Windows, version 12.5 (MedCalc Software, Ostend, Belgium).

## 3. Results

At the end of the study period, 1320 dental implants were placed and a total of 21 different implant systems were used. The distribution of implants by operator is presented in Table 1.

According to the surgeons’ evaluation, 999 curves were suitable and 321 were non-suitable for immediate loading; when evaluated by the AIT, 916 curves belonged to the YES class, while 404 belonged to the NO class. The surgeons and AIT evaluation matched in 1213 cases (91.9%), while they were different in the other 107 occasions (8.1%); in 95 cases, the AIT reported a negative answer while the surgeon considered the curve suitable for immediate loading (7.2%; false negative); in 12 situations, the AIT reported a positive answer while the surgeon considered the curve not-suitable for immediate loading (0.9%; false positive).

**Figure 1:**
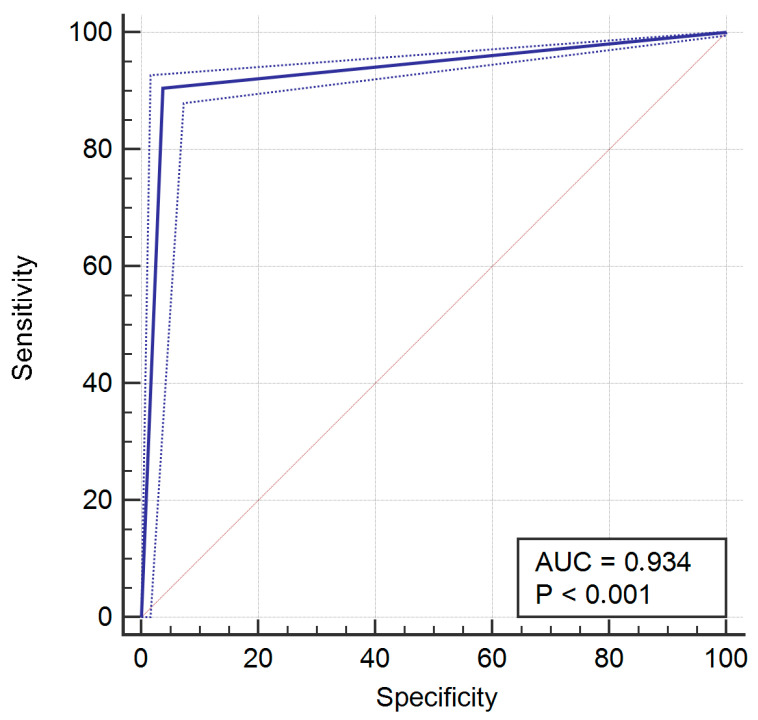
Receiver operating characteristic (ROC) curve for the whole sample.

After analysis, the resulting sensitivity was 90.49% (95% CI = 88.5% to 92.2), specificity was 96.26% (95% CI = 93.6% to 98.1%), PPV was 98.7% (95% CI = 97.7% to 99.3%), and NPV was 76.5% (95% CI = 72% to 80.5%). The ROC for the whole sample is provided in Figure 1. Area under the ROC curve (AUC) was 0.934, with *p* < 0.001.

At the end of the observation period, only four operators placed more than 50 implants (operators 1, 2, 4 and 8) and they were tested individually for the correspondence between their evaluations and those provided by the AIT (Table 2).

For operator 1, a mismatch between evaluations occurred 33 times (6.6%) with 5 (1%) false positives (AIT: YES; operator: not-suitable for immediate loading) and 28 (5.6%) false negatives (AIT: NO; operator: suitable for immediate loading). The resulting sensitivity was 93.4% (95% CI = 90.6% to 95.6%), specificity was 93.42% (95% CI = 85.5% to 97.58%), PPV was 98.8% (95% CI = 97.1% to 99.6%), and was NPV 71.7% (95% CI = 61.8% to 80.3%).

For operator 2, a mismatch between evaluations occurred five times (5.7%), with no false positive and five false negative. The sensitivity was 91.38% (95% CI = 81% to 97.1), specificity was 100% (95% CI = 88.1% to 100%), PPV was 100% (95% CI = 93.3% to 100%), and NPV was 85.3% (95% CI = 68.9% to 95%).

For operator 4, a mismatch between evaluations occurred nine times (16%), with no false positive and nine false negative. The sensitivity was 72.73% (95% CI = 54.5% to 86.7), specificity was 100% (95% CI = 83.9% to 100%), PPV was 100% (95% CI = 85.8% to 100%), and NPV was 70% (95% CI = 50.6% to 85.3%).

For operator 8, a mismatch between evaluations occurred 36 times (7.2%), with 2 (0.4%) false positive and 34 (6.8%) false negative. The sensitivity was 90.53% (95% CI = 87% to 93.4), specificity was 98.57% (95% CI = 94.9% to 99.8%), PPV was 99.4% (95% CI = 97.8% to 99.9%), and NPV was 80.2% (95% CI = 73.5% to 85.9%).

**Figure 2:**
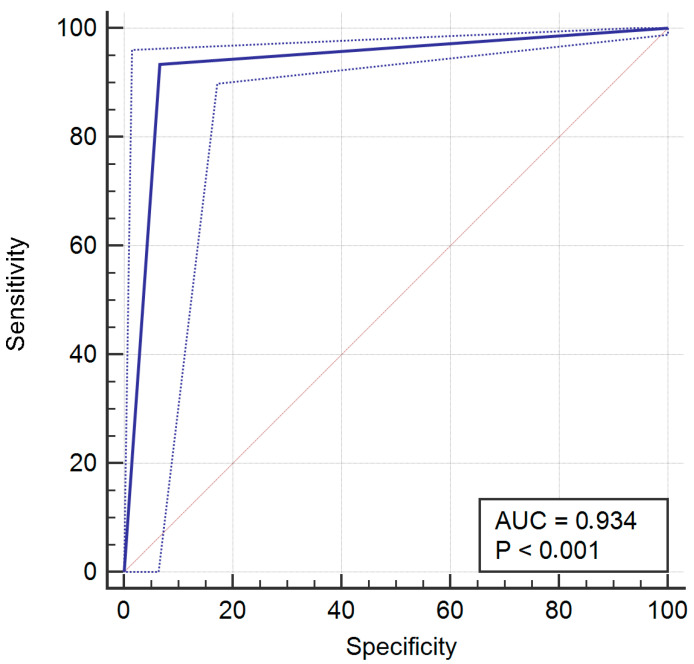
Receiver operating characteristic (ROC) curve for operator 1.

**Figure 3:**
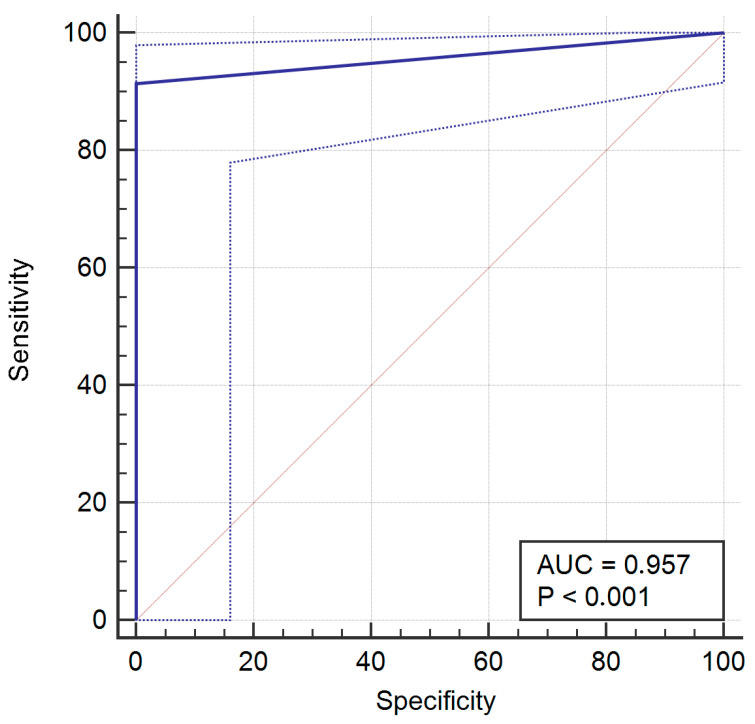
Receiver operating characteristic (ROC) curve for operator 2.

**Figure 4:**
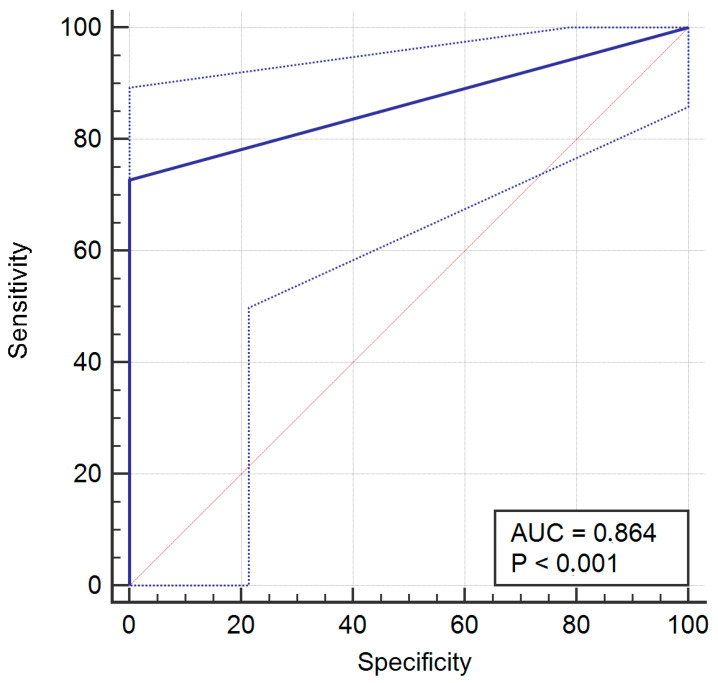
Receiver operating characteristic (ROC) curve for operator 4.

**Figure 5:**
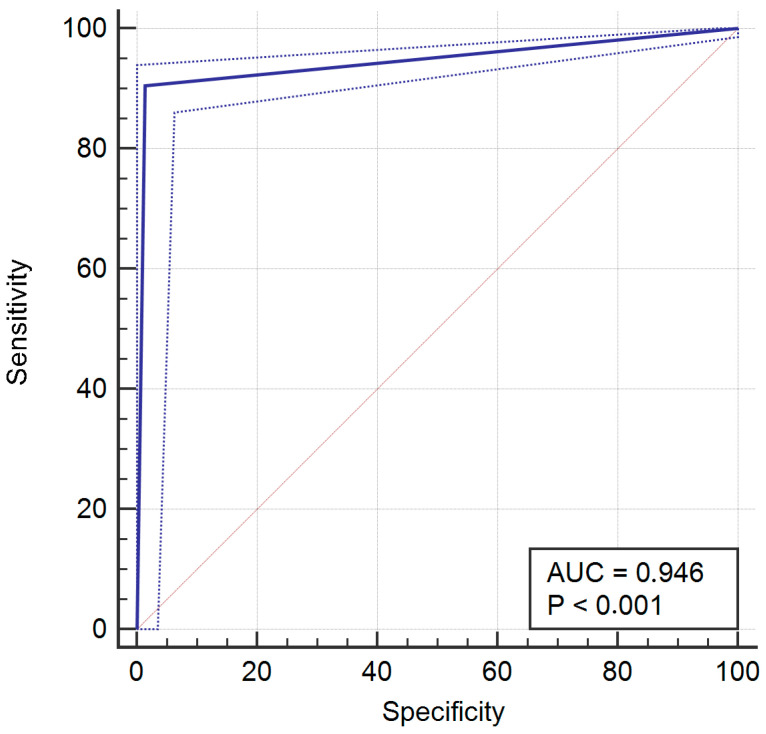
Receiver operating characteristic (ROC) curve for operator 8.

The ROC curves of operators 1, 2, 4, and 8 are provided in Figure 2, Figure 3, Figure 4 and Figure 5.

## 4. Discussion

Primary stability is extremely important for the success of immediately loaded implants, and as a consequence several methods were proposed for its measurement; however, none of them appear effective enough to safely guide a surgeon in the immediate loading feasibility decision. The principal purpose of the present study is to clinically test a recently introduced tool for the assessment of implant primary stability built on artificial intelligence (AI) technology.

The AIT prediction accuracy was quite high, with a sensitivity of 90.49% and a specificity of 96.26%; furthermore, the PPV (98.7%) results show that the AIT very rarely suggested that the surgeon load an implant that did not have appropriate primary stability, while the NPV (76.5%) results express a cautious approach in the determination of immediate loading feasibility.

The main limit of these findings is represented by the difference in terms of implants placed by each single operator; thus, they have to be prudently interpreted to avoid biased conclusions. Nevertheless, when stratified by the operator, the results are very similar and do not seem to be meaningfully influenced by the difference in the number of implants placed.

AI is rapidly expanding in dentistry for numerous purposes [11]; in implant dentistry specifically, an interesting application is its ability to guide an operator in dental implant identification on periapical radiographs [12]. Nevertheless, the tool tested in the present study was only recently presented as a brand-new AI application and, at the moment, no similar methods for the determination of implant primary stability have been presented. Thus, it is impossible to compare the AIT method’s performance with other analogous systems, but it can be useful to evaluate the level of AIT accuracy with other dental applications driven by AI technology.

In an already cited paper about the automated identification of dental implants on periapical radiographs [12], the accuracy in training and validation was very high (99.78% and 99.36%, respectively), but after testing the network performance with previously unknown data, the authors reported a noticeably lower accuracy (85.29%), with a sensitivity of 89.9%, a specificity of 82.4%, PPV of 82.6%, and NPV of 88.5%. Nevertheless, in the conclusions, it was stated that the algorithm provided a high degree of accuracy.

Another recently published paper reported the development and validation of an AI-based system to diagnose gingivitis on intraoral photographs [13]. After the training phase, a validation test was performed comparing the answers of the algorithm to diagnoses performed by a calibrated dentist; the overall sensitivity was 92% and the specificity was 94%. The authors concluded that the system showed a high level of accuracy.

In 2023, Zhu and colleagues [14] presented an AI framework developed to diagnose multiple dental diseases on panoramic radiographs; specifically, the framework was instructed to recognize caries, residual roots, impacted teeth, full crowns, and missing teeth. After the training phase, the sensitivity and specificity were, respectively, 96.4% and 99.6% for impacted teeth, 95.3% and 99.8% for full crowns, 87.1% and 99.9% for residual roots, 88.5% and 99.4% for missing teeth; and 55.4% and 99% for caries. The diagnostic performance was then compared with the diagnoses of nine dentists with three different levels of clinical experience (more than 10 years, between 3 and 10 years, or less than 3 years of seniority in the stomatology hospital). The accuracy of the AI framework was generally high, even if it was lower than the evaluation accuracy of some of the more experienced dentists in diagnoses of impacted teeth, missing teeth, and caries especially. By consequence, the authors concluded that “caries diagnosis by the AI framework remained a challenge”.

Finally, a paper published in 2023 [15] showed the results obtained by an AI-driven system for the detection of dental formula and the presence of dental implants, prosthetic crowns, fillings, root canal treatments, and root remnants. After the evaluation of the diagnostic performance by two experienced clinicians, the overall sensitivity was 89%, the overall specificity was 98%, the PPV was 94%, and the NPV was 97%. Nevertheless, the performance of the algorithm changed depending on the object of detection; in particular, the authors showed the best values for the correct identification of the presence of the teeth and crowns (sensitivity > 90%) and the worst values for the correct detection of fillings and residual remnants (sensitivity < 70%), with a specificity always >90%.

The comparison of the AIT in this study’s sensitivity, specificity, PPV, and NPV to those of other AI-driven dental applications recently presented in peer reviewed papers shows that the AIT’s accuracy is high and in line with published AI systems’ standards. These results are particularly interesting considering the number of surgeons involved, the variety of implant systems, and the absence of limitations in terms of implant dimensions, bone quality, and implantation timing.

Artificial intelligence has been used in various industries for a long time already, and its applications in dentistry are increasing, aiding operators in their daily clinical choices. The results of the present paper seem to prove once again the potential power of AI. Nevertheless, it is important to remember that AI can be a valid support as long as the final clinical decisions are reserved for dental professionals and its applications meet the principles stated in the WHO guidance [16]. This analysis of the outcomes is extremely encouraging for AIT development. This new tool seems to be able to evaluate the insertion torque curve trend with a very high level of precision and thus aids dentists in the very difficult decision of immediate loading feasibility. Furthermore, the small number of false positives leads to a very cautious approach to the suggestions provided by AIT, and this is extremely important, especially for immediately loaded single crowns. Clinical applications of the results could be particularly beneficial for unexperienced operators that want to approach immediate loading techniques, and the technology could also be a valid aid for dentists facing challenging clinical situations.

## 5. Conclusions

The AIT tested in the present clinical multicenter study demonstrated a high level of accuracy in the prediction of immediate loading feasibility. Even in the presence of differences in terms of number of implants placed by each single operator, the results seem to suggest that AIT can be a useful aid for clinicians in immediate loading decisions, regardless of implant system used or the clinical condition treated.

## Figures and Tables

**Table 1:** Distribution of implants inserted by operator.

Operator	Number of Implants	Percent of the Whole Sample
1	500	37.8
2	87	6.6
3	9	0.7
4	54	4.1
5	22	1.7
6	40	3
7	19	1.5
8	499	37.8
9	25	1.9
10	45	3.4
11	20	1.5

**Table 2:** Sensitivity, specificity, PPV, and NPV of the entire sample and stratified by operators with more than 50 implants placed.

Number of Implants	Sensitivity	Specificity	PPV	NPV
1320	90.49%	96.26%	98.7%	76.5%
500	93.4%	93.42%	98.8%	71.7%
87	91.38%	100%	100%	85.3%
54	72.73%	100%	100%	70%
499	90.53%	98.57%	99.4%	80.2%

## Data Availability

The data presented in this study are available on request from the corresponding author.
